# Clinical application of the femoral neck system in the treatment of femoral neck fractures

**DOI:** 10.3389/fsurg.2026.1722845

**Published:** 2026-01-27

**Authors:** Guangyao Li, Xiaodan Xu, Junlong Song, Jinbo Liu, Qingsong Li, Zhenhai Pan, Weize Sun, Jingri Jin

**Affiliations:** 1 Medical College of Yanbian University, Yanbian, Jilin, China; 2 Department of Anesthesiology, Tai'an Eightty-Eight Hospital, Tai'an, Shandong, China; 3 Orthopedic Diagnosis and Treatment Center, Yanbian University Hospital, Yanbian, Jilin, China; 4 Department of Orthopedics, Traditional Chinese Medicine Hospital of Antu County, Yanbian, Jilin, China; 5 Joint Surgery Department, Tai'an Eightty-Eight Hospital, Tai'an, Shandong, China

**Keywords:** cannulated compression screw, femoral neck fracture, femoral neck system, internal fixation, young and middle-aged patients

## Abstract

**Objective:**

The research was conducted to assess the therapeutic efficacy of the femoral neck system (FNS) and cannulated compression screw (CCS) in treating femoral neck fractures classified as Pauwels type II and III in young to middle-aged patients.

**Methods:**

This retrospective cohort study included 46 patients treated at Yanbian University Affiliated Hospital between January 2021 and March 2023. Patients were allocated into two groups: FNS (*n* = 24) and CCS (*n* = 22). Clinical indicators, including bone healing duration, Visual Analog Scale (VAS), Harris Hip Score (HHS), and postoperative complications, were compared.

**Results:**

Satisfactory fracture reduction was consistently accomplished in all individuals, with observation continuing over 9 to 24 months. Baseline characteristics were comparable across both groups. The FNS group showed a significantly shorter healing time (4.00 ± 1.00 vs. 4.79 ± 1.39 months, *P* < 0.05), and higher HHS at 3 and 6 months following surgery (*P* < 0.05). VAS scores and 1-month HHS showed no significant differences between groups. Femoral neck shortening (4.2% vs. 27.3%, *P* = 0.043) and hardware failure (4.2% vs. 31.8%, *P* = 0.020) were significantly less frequent in the FNS group, while rates of femoral head necrosis (4.2% vs. 9.1%) and nonunion (12.5% vs. 13.6%) were comparable.

**Conclusion:**

FNS promotes faster fracture recovery, reduces fixation-related complications, and improves early hip function, making it a reliable and efficient option for surgical stabilization of femoral neck fractures in young and middle-aged populations.

## 1 Introduction

Femoral neck fractures (FNF) are among the most common types of hip fractures in elderly individuals, typically arising from low-energy trauma. In contrast, FNF occurring in individuals aged 18 to 65 are typically triggered by incidents like car crashes or vertical falls (1, 2). It has been reported that FNF ccount for about 4% of all adult fractures and nearly 48% of hip-related injuries in China. Among these, approximately 3% of hip fractures occur in young and middle-aged individuals (3, 4). By 2050, the global occurrence rate of FNF is projected to reach 6.3 million cases, with over 50% occurring in Asia. As a populous country, China faces substantial economic burdens on both society and families due to its high costs of treatment. This poses a growing public health concern and a significant challenge for orthopedic surgeons (5, 6).

The majority of patients with FNF require surgical treatment, and the treatment approach should be determined based on elements like the anatomical position where the fracture occurs, extent of displacement, and general health condition of the patient. For elderly patients, hip arthroplasty is generally the preferred option to avoid reoperation and the complications caused by prolonged bed rest after internal fixation (7). However, for young patients with FNF, internal fixation aimed at preserving the femoral head is preferred due to factors such as fracture healing potential, life expectancy, the demand for hip joint function, and the lifespan of the prosthesis (8). Regardless of whether open reduction or closed reduction is performed, the rate of complications following surgery remains high, encompassing femoral neck collapse, necrosis of the femoral head, and nonunion. Slobogean et al. conducted a study analyzing 1,558 patients with FNF who underwent internal fixation. They uncovered that 14.3% of patients developed femoral head necrosis following surgery, while the rate of nonunion was 9.3% (2).

Currently, cannulated compression screw (CCS) remains the dominant modality used to stabilize FNF in China. The traditional CCS involves the use of three parallel CCSs to fix the fracture site. It has the function of dynamic sliding compression, which can dynamically compress the fracture site and promote fracture healing. Additionally, compared to other fixation methods, CCS can lead to less trauma, reduced blood loss, and shortened operative time. Therefore, for a prolonged period, CCS has been the preferred method for the internal fixation of FNF among many clinicians (9, 10). However, according to relevant studies, in FNF with a larger Pauwels angle, CCS may result in issues including femoral neck collapse and implant failure caused by inadequate resistance and poor angular control, with a surgical failure rate as high as 43% (10, 11).

The femoral neck primarily experiences stress in two directions: one is the stress aligned with the longitudinal axis of the femoral neck that compresses the neck. While this stress is beneficial for fracture healing, it can also lead to issues including loss of fixation integrity and femoral neck collapse. The second is the stress perpendicular to the femoral neck's anatomical axis. This stress can generate shear force at the fracture site. Additionally, a larger Pauwels angle can lead to a greater shear force, thereby causing a higher incidence of complications. Therefore, an ideal internal fixation device must provide support for the stress in these two directions (12).

In recent years, a novel internal fixation device developed by DePuy Synthes—the femoral neck system (FNS)—has been introduced, consisting of a dynamic rod, a plate, an anti-rotation screw, and a locking screw. Biomechanical research has demonstrated that the FNS offers superior angular stability as well as torsional stability in contrast to the CCS (13–15). Nevertheless, the advantages of applying FNS over traditional CCS for FNF treatment remain controversial, given the short duration of clinical implementation. Although there are some mechanical and theoretical benefits, limited research has reported on the clinical outcomes of FNF. Moreover, the results of these studies are inconsistent, particularly regarding intervention outcomes among young and middle-aged individuals.

Therefore, this study retrospectively analyzed clinical data of patients younger than 65 years with FNF treated with FNS or CCS at Yanbian University Affiliated Hospital between January 2021 and March 2023. The clinical efficacy and incidence of complications were compared, providing evidence-based medical data and references for the treatment strategies of FNF.

## 2 Materials and methods

### 2.1 Clinical data

Clinical data from 62 FNF patients were retrospectively examined for those who underwent internal fixation treatment from January 2021 to March 2023. According to the defined eligibility criteria, 46 cases meeting the requirements were selected, all of whom had a postoperative follow-up period of more than 9 months. Participants were stratified into two cohorts according to treatment modality: FNS-treated patients (*n* = 24) and those treated with CCS (*n* = 22). Because this was a retrospective cohort study, patients were not randomly assigned to the FNS or CCS groups. The choice of fixation method was determined by the treating surgeon based on routine clinical practice and implant availability at the time of treatment. Therefore, this study adopted a non-randomized retrospective grouping design, and the possibility of selection bias cannot be completely excluded. As this was a retrospective observational study, no *a priori* sample size calculation was performed. Approval was granted by Ethics Committee of Affiliated Hospital of Yanbian University (APproval No.2025140).Written informed consent was obtained from all participants prior to their inclusion in the study.The study was conducted in accordance with the principles of the Declaration of Helsinki.

Eligibility criteria included: (1) patients aged 18 to 65 years; (2) diagnosis of unilateral, closed FNF; (3) fracture type classified based on preoperative imaging as Pauwels type II (FNF angle between 30° and 50°, representing a moderate angle fracture with relatively stable fracture lines and a favorable prognosis for healing) or type III (FNF angle greater than 50°, typically more unstable, with increased difficulty in healing and a higher risk of femoral head necrosis) (16); (4) normal hip joint function before the injury, with no underlying diseases affecting hip joint mobility; and (5) postoperative follow-up of at least 9 months, with complete case data available.

The criteria for exclusion included (1) individuals presenting with pathological bone lesions caused by tumors or other diseases; (2) Individuals with old FNF (injury occurring more than 3 weeks prior); (3) Individuals with multiple injuries; (4) Individuals with a prior history of long-term alcohol overuse, prolonged hormone use, or pre-existing femoral head necrosis; (5) Individuals diagnosed with psychiatric conditions that impaired compliance with postoperative follow-up and reviews; and (6) patients who were lost or died during the follow-up period.

### 2.2 Methods

#### 2.2.1 Pre-surgical procedures

All subjects meeting the inclusion criteria underwent pelvic x-rays and physical examinations preoperatively. If necessary, a three-dimensional computed tomography (CT) scan targeting the injured hip was conducted to verify the diagnosis and fracture type. To ensure accurate Pauwels classification, all patients underwent standard anteroposterior and lateral hip radiographs, which serve as the primary and widely accepted basis for measuring fracture inclination. Three-dimensional CT was performed when the fracture line could not be clearly delineated on plain radiographs, following our routine clinical workflow. CT was also performed in patients with suspected posterior or cortical comminution to better evaluate fracture morphology. Patients with severely comminuted fractures that were unsuitable for internal fixation were excluded according to the study criteria. MRI was reserved for cases in which occult fractures or femoral head pathology were suspected. The indications for additional CT/MRI were applied uniformly across both groups, minimizing any impact of imaging heterogeneity on Pauwels classification. Routine preoperative tests included chest x-rays or chest CT, electrocardiograms, and relevant blood tests. Additional imaging, such as magnetic resonance imaging (MRI) or ultrasound, was performed as needed based on the patient's medical history for comprehensive preoperative evaluation. The surgical risks and potential complications were communicated to the patient, and approval was formally received from both the patient and their family for the proposed treatment plan. Patients were instructed to fast for 8 h and refrain from drinking fluids for 4 h prior to surgery. Additionally, antibiotic administration via intravenous drip was given 30 min before surgery to prevent infection.

#### 2.2.2 Surgical method

Closed reduction was first achieved on a traction table through longitudinal traction with gentle internal rotation, and its quality was preliminarily confirmed under C-arm fluoroscopy. Supine positioning on the traction table was achieved under general or regional anesthesia. Then, anteroposterior and lateral images were taken using the intraoperative C-arm. The contralateral limb was arranged in hip flexion, knee flexion, and abduction and fixed on the traction table. The operative limb was kept in a neutral alignment with adequate traction and internal adduction and rotation. After satisfactory reduction based on the Garden alignment assessment under the C-arm, the limb was fixed. The surgical area was then routinely disinfected, and sterile drapes were applied.

**Figure 1:**
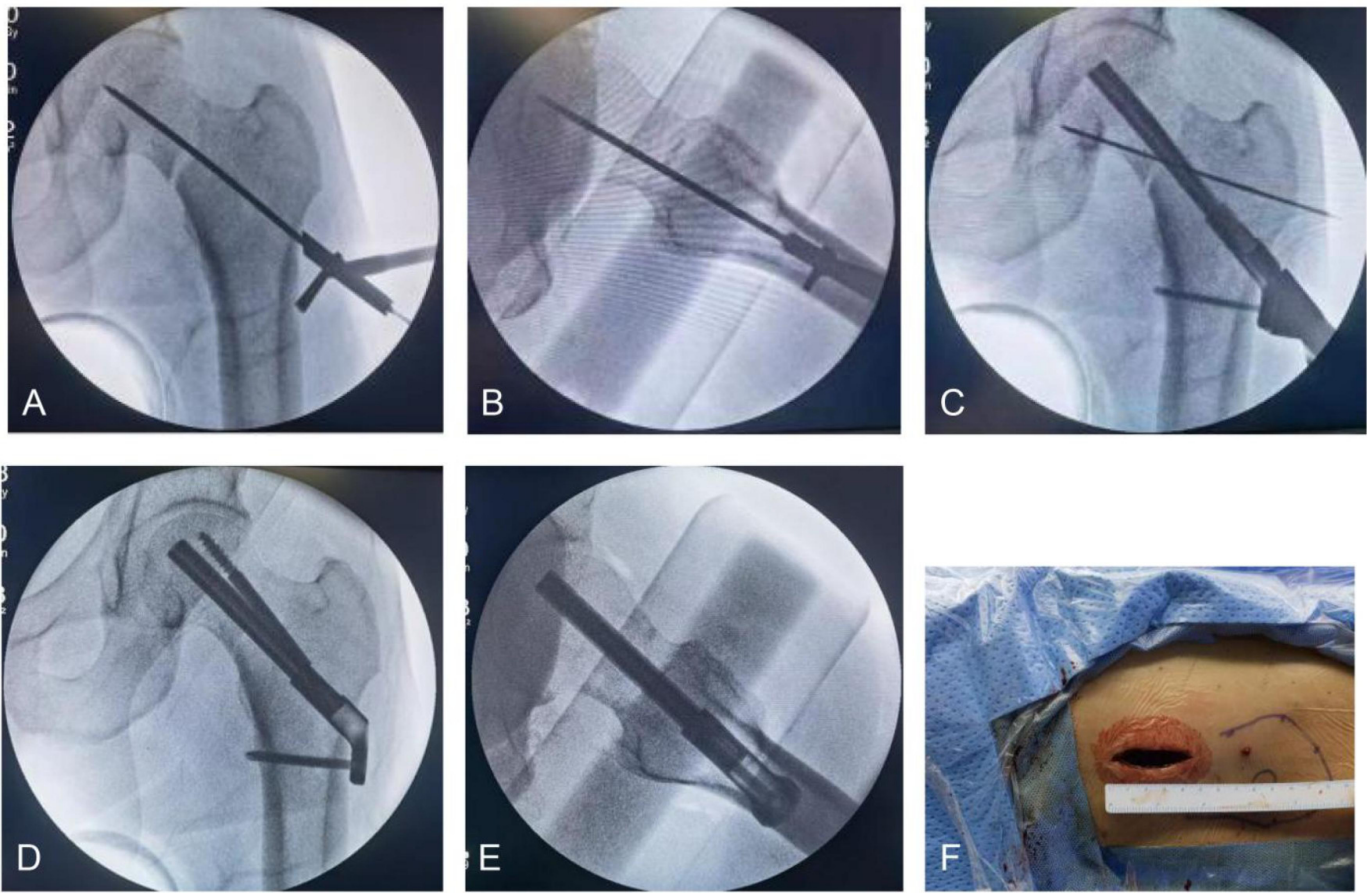
**(A,B)** Insertion of the guidewire under the guidance of the guide; **(C)**, after drilling, insertion of the dynamic rod and distal locking screws; **(D,E)**, compression of the fracture site after insertion of the anti-rotation screw; F, incision length of approximately 4 cm.

Subsequently, the FNS group underwent the following procedure: With C-arm fluoroscopic guidance, a 2.0 mm Kirschner wire was introduced along the femoral neck axis to prevent rotational displacement of the fracture site, with the wire positioned anterosuperiorly within the femoral neck. Thereafter, a 4 cm longitudinal incision was created at the level corresponding to the lesser trochanteric area, below the greater trochanter, and the iliotibial band along with the lateral vastus was separated to visualize the periosteum. At this level, a guidewire was placed via a 130° orientation tool, verifying that the guidewire was tightly attached to the femur. The guidewire position was centered in both anteroposterior(AP) and lateral views, with its tip located 5 mm proximal to the subchondral bone. After measuring the depth, the hole was reamed over the central wire, then a locking plate and dynamic rod were positioned. The position of the locking plate was then confirmed by palpation and the C-arm, ensuring that it was aligned along the midline of the femoral shaft and in close contact with the lateral cortex of the femur. Using the surgical guide tool, stabilizing and anti-torsion screws were inserted. The length of the anti-rotation screws was identical to that of the dynamic rod. After removing the 2.0 mm Kirschner wire, compression at the fracture site was achieved under C-arm fluoroscopy by rotating the multifunctional rod counterclockwise. Once the reduction quality was reconfirmed as satisfactory and the internal fixation was properly positioned under the C-arm, the surgical site was irrigated, and the incision was closed in layers. The entire surgical process is shown in Figure 1, which illustrates guidewire insertion (A–B), placement of the dynamic rod and locking screws (C), fracture compression after anti-rotation screw placement (D–E), and the approximately 4 cm surgical incision (F).

In CCS-assigned cases, the treatment approach was as follows: A 4 cm incision was performed at the lesser trochanter level, below the greater trochanter. Under the C-arm, a guidewire was inserted parallel to the femoral neck axis, positioned above the femoral head, and advanced to a depth of 5 mm from the subchondral interface. Under the guidance of a parallel guide, additional guidewires were inserted at the anterosuperior and posterosuperior regions of the femoral neck, forming an inverted three-point construct. After measuring the depth, the hole was reamed over the guidewires, after which three cannulated screws were inserted one by one. After confirming satisfactory positioning under the C-arm, the three guidewires were removed. The surgical site was irrigated, and the incision was closed in layers.

#### 2.2.3 Postoperative management

Postoperatively, patients were given symptomatic treatment, such as electrocardiographic monitoring, analgesia, and rehydration. Antibiotics were administered within 24–48 h postoperatively to prevent infection. To prevent the onset of lower extremity thrombosis, oral rivaroxaban (10 mg once daily) was administered from the first postoperative day and continued for 4 weeks. On the first postoperative day, the affected hip was reviewed using the x-ray, and the patient was instructed to perform functional exercises while in bed, such as ankle pump exercises, quadriceps isometric contractions, and passive knee flexion and extension. On the third postoperative day, patients were allowed to perform non-weight-bearing ambulation with bilateral crutches. Follow-up visits were scheduled at approximately 1, 2, 3, and 6 months postoperatively. The exact dates of outpatient examinations were recorded automatically in the electronic medical record. The timing of limited and full axial loading was determined based on imaging findings and physical examination results. In our institution, partial weight-bearing was allowed when radiographs showed early callus formation without secondary displacement, whereas full axial loading was permitted only after cortical continuity and progressive blurring of the fracture line were observed. These decisions were based on routine clinical judgment rather than quantitative radiologic scoring and were applied consistently to both the FNS and CCS groups, ensuring that postoperative weight-bearing progression did not differ between groups. Fracture healing time was defined as the interval between surgery and the first follow-up radiograph demonstrating cortical continuity and disappearance or marked blurring of the fracture line. For the calculation of healing time, the interval between the date of surgery and the actual calendar date of the confirming radiograph was extracted and expressed in months. Healing time was analyzed as a continuous variable. If any clinical changes were observed, the follow-up schedule was adjusted accordingly.

### 2.3 Evaluation indicators

This study conducted a comparison of preoperative, intraoperative, and postoperative data across both cohorts. Preoperative data comprised body mass index (BMI), age, gender, injury-to-surgery time, injury etiology, and Pauwels classification. Among them, the fracture mechanisms consisted of low-energy and high-energy trauma, and the Pauwels classification was limited to type II or type III fractures. The intraoperative data involved the incision length, surgical time, and fracture reduction precision (as per Garden alignment assessment). In order to assess the reduction quality of FNF, x-rays of the involved femoral neck captured in both AP and lateral views under the C-arm were used, and the Garden alignment index (17) was applied as the standard for reduction assessment. The standard anteroposterior x-ray shows a 160-degree angle between the medial margin of the femoral shaft and the main compressive trabeculae of the femoral head, whereas the lateral projection illustrates a 180° alignment between the axes of the femoral head and neck. An unsatisfactory reduction was defined as an AP angle <155° or lateral angle >180°, which may increase the probability of femoral head necrosis. The reduction was categorized into four grades based on the Garden alignment criteria: Grade I (160°AP, lateral 180°); Grade II (155° AP, lateral 180°); Grade III (AP < 155°, lateral >180°); Grade IV (150° AP, lateral >180°). Postoperative data included fracture union duration, the rate of postoperative adverse events, including shortening of the femoral neck, osteonecrosis, nonunion, and screw loosening or breakage, Visual Analog Scale (VAS) score, and Harris Hip Score (HHS).

The criteria for fracture healing were displayed as follows: (1) no tenderness or longitudinal percussion pain at the affected hip; (2) no abnormal localized mobility; and (3) the presence of a blurred fracture line and continuous bone callus formation on x-ray. The HHS consists of four components: pain, functional capacity, anatomical deformity, and joint mobility, based on a maximum score of 100 points. Grading was as follows: excellent (≥90), good (80–89), moderate (70–79), and poor (<70). The measurement of femoral neck shortening followed the protocol proposed by Slobogean et al. (18). Briefly, based on pelvic AP x-ray, the shortening was calculated as the total bilateral discrepancy in the distance from the medial aspect of the femoral head to the outer limit of the greater trochanter, alongside differences in apex-to-apex alignment. Overall, the sum of the measurement differences in the horizontal and vertical directions was used as the femoral neck shortening value.

### 2.4 Statistical analysis

For statistical processing, SPSS Statistics 26.0 (IBM) was employed. Normality of continuous variables was assessed using the Shapiro–Wilk test. Measurement data conforming to a normal distribution were expressed as mean ± standard deviation and compared between groups using independent-samples *t*-tests. For non-normally distributed data, the Mann–Whitney *U*-test was applied. Categorical variables were presented as frequencies or percentages and analyzed using the chi-square test or Fisher's exact test when appropriate. A two-sided *P* value <0.05 was considered statistically significant.

## 3 Results

### 3.1 Preoperative patient profile comparison

A sample of 46 individuals was allocated into two groups depending on the treatment modality: FNS (*n* = 24) and CCS (*n* = 22). The FNS group consisted of 12 males and 12 females (mean age: 50.21 ± 11.23 years), while the CCS group included 15 males and 7 females (mean age: 48.00 ± 10.34 years). No statistically meaningful differences were detected across age, body mass index, gender, and injury-to-surgery interval across the FNS and CCS groups (*P* > 0.05, Table 1).

**Table 1:** Preoperative demographic and clinical data of FNS vs. CCS groups.

Research Indicator	FNS (*n* = 24)	CCS (*n* = 22)	t/*χ*²	*P*
Age (years)	50.21 ± 11.23	48.00 ± 10.34	0.692	0.493
BMI (kg/m²)	21.79 ± 2.89	22.53 ± 2.77	−0.950	0.347
Gender (Male/Female)	12/12	15/7	1.565	0.211
Time from injury to surgery (days)	2.42 ± 2.36	2.45 ± 2.97	−0.048	0.962

BMI, body mass index.

Regarding the fracture mechanism, low-energy trauma was the primary mechanism in both groups. Falls were considered low-energy trauma, whereas high-energy trauma included both traffic-related incidents and height-related falls. Within the FNS group, 8 cases (33.3%) were attributed to high-energy trauma, and 16 (66.7%) to low-energy. In comparison, the CCS group showed 5 high-energy (22.7%) and 17 low-energy events (77.3%). The intergroup variation did not reach statistical significance (*P* > 0.05). Regarding Pauwels classification, in the FNS group, 15 cases (62.5%) were classified as Pauwels type II and nine cases (37.5%) as Pauwels type III. In the CCS group, 14 cases (63.6%) were categorized as Pauwels type II and eight cases (36.4%) as Pauwels type III. No noteworthy variation emerged across the FNS vs. CCS cohorts (*P* > 0.05; Table 2). In summary, this indicates homogeneity in baseline features, permitting valid between-group comparison.

**Table 2:** Injury mechanism and Pauwels classification: FNS vs. CCS.

Research indicator	FNS (*n* = 24)	CCS (*n* = 22)	χ²	*P*
Injury mechanism				
High-energy trauma	8 (33.3%)	5 (22.7%)	0.637	0.425
Low-energy trauma	16 (66.7%)	17 (77.3%)		
Pauwels classification				
Type Ⅱ	15 (62.5%)	14 (63.6%)	0.006	0.936
Type Ⅲ	9 (37.5%)	8 (36.4%)		

### 3.2 Comparison of intraoperative general data

The average surgical time and incision length in the FNS group were 73.33 ± 19.15 min and 4.04 ± 0.75 cm, respectively, while in the CCS group, these values were 73.64 ± 22.42 min and 4.09 ± 0.92 cm, respectively. There were no statistically meaningful differences between the FNS and CCS groups (*P* > 0.05). According to the Garden alignment index, 20 cases were grade I and 4 were grade II in the FNS group, while 18 cases were grade I and 4 were grade II in the CCS group, with no significant difference between groups (*P* > 0.05; Table 3).

**Table 3:** Comparison of surgical time, incision length, and garden alignment index: FNS vs. CCS.

Research indicator	FNS (*n* = 24)	CCS (*n* = 22)	Statistic	*P*
Surgical time (minutes)	73.33 ± 19.15	73.64 ± 22.42	−0.049	0.961
Incision length (cm)	4.04 ± 0.75	4.09 ± 0.92	−0.199	0.843
Garden alignment index				
Ⅰ	20	18	-	1.000a
Ⅱ	4	4		

“a” indicated Fisher's exact test; “-” indicates that the statistic is not applicable.

### 3.3 Comparison of postoperative fracture healing time and VAS scores

In the FNS group, fracture consolidation occurred sooner (4.00 ± 1.00 months) relative to the CCS group (4.79 ± 1.39 months) (*P* = 0.045). However, VAS score at both 1 and 3 months postoperatively showed no intergroup significance (*P* > 0.05; Table 4).

**Table 4:** Analysis of post-surgical fracture healing interval and VAS scores: FNS vs. CCS.

Research indicator	FNS (*n* = 24)	CCS (*n* = 22)	t	*P*
Fracture healing time (months)	4.00 ± 1.00	4.79 ± 1.39	−2.070	0.045
VAS score at 1 month postoperatively	3.96 ± 0.91	4.27 ± 0.88	−1.189	0.241
VAS score at 3 months postoperatively	2.58 ± 0.72	2.77 ± 0.87	−0.809	0.423

VAS, visual analog scale.

### 3.4 Comparison of postoperative hip joint function

Evaluation of hip function was conducted with the HHS at 1-, 3-, and 6-month follow-ups. At 3 and 6 months, the HHS in the FNS group (72.63 ± 5.73 and 81.75 ± 4.21, respectively) was significantly higher than that in the CCS group (69.00 ± 6.29 and 78.36 ± 5.65, respectively) (*P* < 0.05), while no appreciable intergroup distinction was observed at 1 month (*P* > 0.05; Table 5). These results suggested that FNS demonstrates favorable efficacy in promoting early hip joint functional recovery.

**Table 5:** Comparison of postoperative hip joint function: FNS vs. CCS.

HHS	FNS (*n* = 24)	CCS (*n* = 22)	t	*P*
1 month postoperatively	42.01 ± 2.65	40.97 ± 2.56	1.328	0.191
3 months postoperatively	72.63 ± 5.73	69.00 ± 6.29	2.046	0.047
6 months postoperatively	81.75 ± 4.21	78.36 ± 5.65	2.316	0.025

HHS, harris hip score.

### 3.5 Comparison of postoperative complications

**Figure 2:**
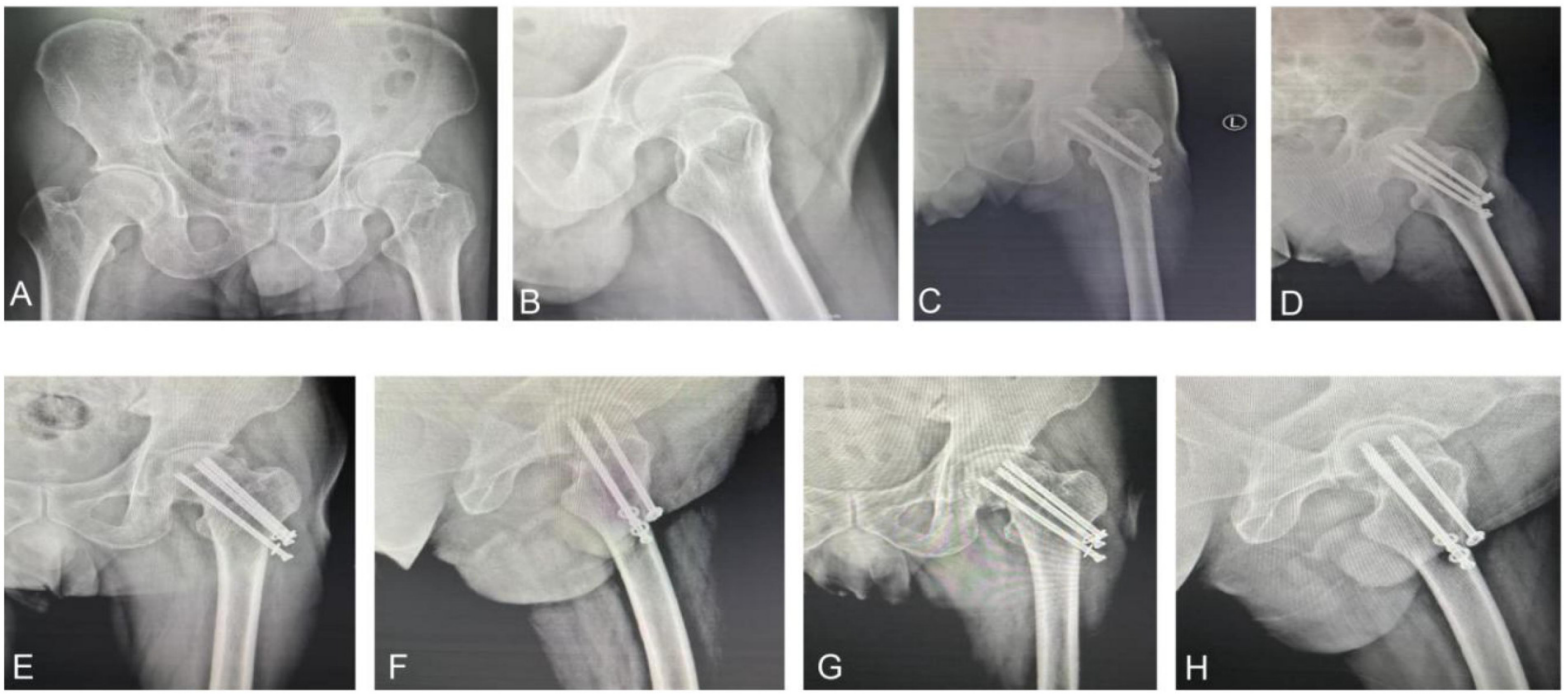
A 58-year-old case sustained a Pauwels type II FNF managed by CCS within 24 h subsequent to a fall. **(A,B)** Pre-op pelvic radiographs: AP **(A)** and lateral **(B)** views; **(C,D)** AP **(C)** and lateral **(D)** views on day 1 postoperatively; **(E,F)** AP **(E)** and lateral **(F)** at 3 months postoperatively, demonstrating loosening at the distal cannulated screw; **(G,H)** AP **(G)** and lateral **(H)** images at 6 months postoperatively, revealing complete loosening of all three screws with femoral neck shortening.

During the follow-up period, a single FNS patient (4.2%) exhibited femoral neck shortening, whereas six patients (27.3%) in the CCS group experienced this complication, yielding a significant intergroup difference (*P* = 0.043). A representative case from the CCS group is presented in Figure 2, demonstrating progressive implant loosening and femoral neck shortening from the immediate postoperative period to 6 months after surgery. Additionally, screw loosening or breakage occurred in 1 patient (4.2%) in the FNS group and 7 patients (31.8%) in the CCS group, the incidence of screw loosening or breakage was markedly reduced in the FNS group (4.2%) in contrast to the CCS group (31.8%), demonstrating statistical significance (*P* = 0.020). Both groups exhibited similar outcomes regarding femoral head necrosis (1/24, 4.2% vs. 2/22, 9.1%) and nonunion (3/24, 12.5% vs. 3/22, 13.6%), with no significant differences (*P* > 0.05; Table 6).

**Table 6:** Postoperative events in FNS vs. CCS patients.

Postoperative complications	FNS (*n* = 24)	CCS (*n* = 22)	*P*
Femoral neck shortening	1 (4.2%)	6 (27.3%)	0.043
Internal fixation failure (Loosening/Breakage)	1 (4.2%)	7 (31.8%)	0.020
Femoral head necrosis	1 (4.2%)	2 (9.1%)	0.600
Nonunion	3 (12.5%)	3 (13.6%)	1.000

**Figure 3:**
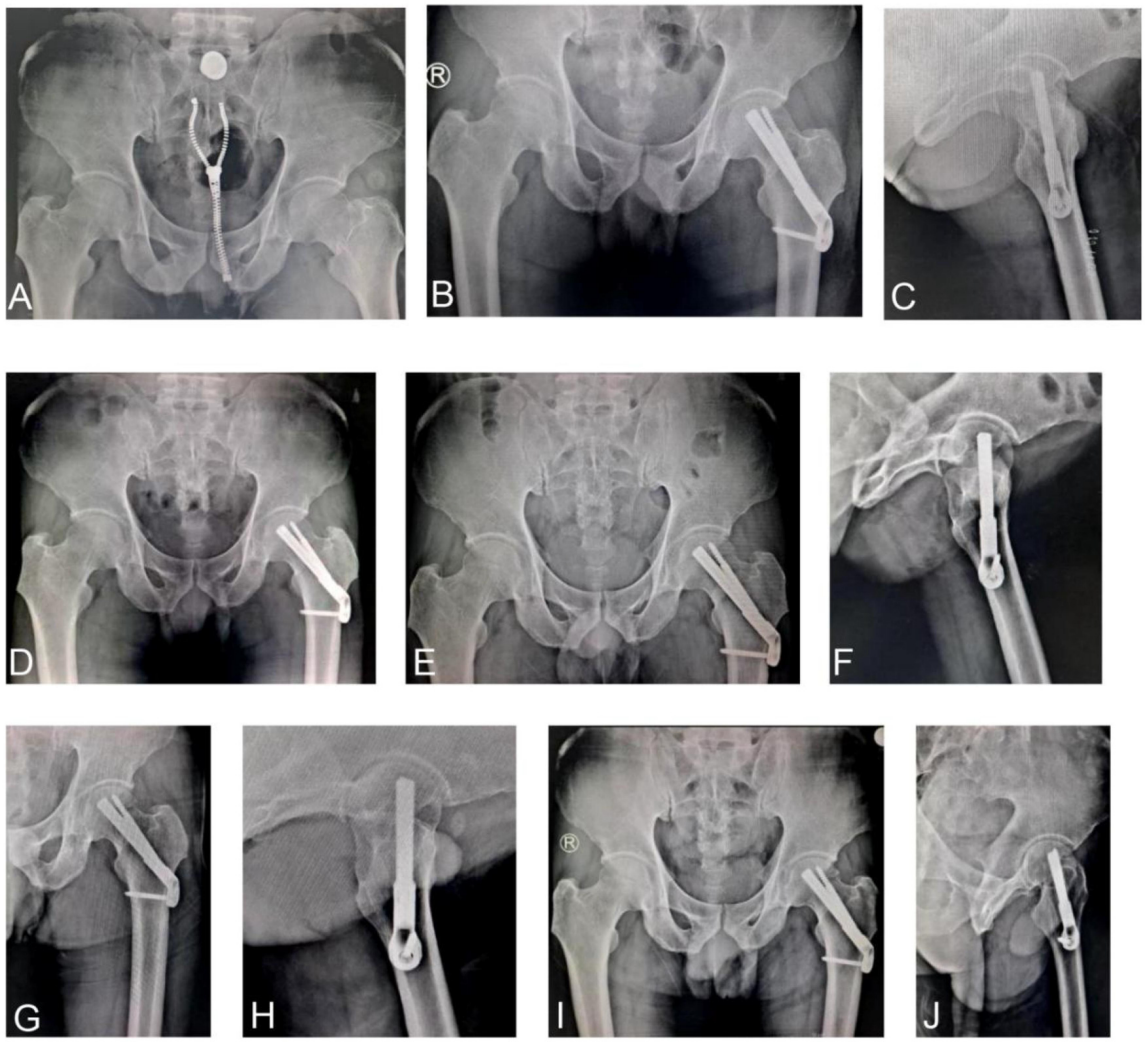
A 56-year-old patient sustained a Pauwels type III FNF following a fall and underwent FNS 24 h after injury. **(A)**, Pre-surgical pelvic x-ray in AP view; **(B,C)**, AP **(B)** and lateral **(C)** views on day 1 postoperatively; **(D)**, Anteroposterior x-ray at 1 month postoperatively; **(E,F)**, AP **(E)** and lateral **(F)** x-rays obtained 3 months after surgery, showing a blurred fracture line; **(G,H)**, AP **(G)** and lateral **(H)** x-rays at 6 months postoperatively, revealing complete fracture healing; **(I,J)**, AP **(I)** and lateral **(J)** x-rays at 12 months postoperatively.

To further illustrate the healing progression observed in the FNS group, Figure 3 presents the serial preoperative and follow-up radiographs of a representative Pauwels type III case treated with FNS, demonstrating stable fixation, gradual blurring of the fracture line by three months, complete healing at six months, and continued remodeling at twelve months.

## 4 Discussion

This study explored the therapeutic differences between FNS and CCS among individuals aged 18–65 years diagnosed with Pauwels type II and III FNF. The results demonstrated that FNS achieved shorter fracture healing time, better hip function recovery, and lower rates of fixation-related complications compared with CCS. Notably, FNS demonstrated a significant advantage in reducing neck shortening and loss of implant stability. The superior performance of FNS may be explained, at least in part, by its improved shear resistance and rotational stability, thereby potentially conferring greater biomechanical strength in unstable fractures and contributing to a reduced rate of postoperative complications.

In recent years, the occurrence rate of FNF among individuals aged 18 to 65 has been rising, attributed to an increase in high-energy trauma, particularly Pauwels type II and III fractures (19, 20). However, these fractures are prone to surgery-related complications like femoral neck shortening and failed fixation, due to significant vertical shear forces. Given its superior rotational stability and minimal trauma, the FNS is increasingly employed in addressing FNF cases (21–24). For Pauwels type FNF, CCS demonstrates insufficient stability in shear resistance, making it prone to postoperative screw loosening and femoral neck shortening (25, 26). In contrast, FNS appears to help prevent these complications, promote fracture healing, and improve hip joint function through its dynamic rod and locked compression mechanism (14, 27). Given the biomechanical differences between Pauwels II and III fractures, some heterogeneity in treatment outcomes may exist. Pauwels III fractures are more vertically oriented and inherently unstable, making them theoretically more likely to benefit from the superior angular and rotational stability of the FNS. However, the sample size in each subgroup was relatively small, and further stratified analysis would markedly reduce statistical power and increase the likelihood of type II error. Moreover, the distribution of Pauwels classifications was well balanced between the FNS and CCS groups, minimizing potential confounding. Larger multicenter studies are warranted to clarify whether the clinical advantage of FNS differs between Pauwels II and III fractures.

In this study, the two groups did not differ significantly in preoperative general data. However, regarding the injury mechanism, each group primarily presented with low-energy trauma. Among the 46 patients, 33 cases (71.7%) were caused by low-energy trauma, for example, falls, whereas 13 cases (28.3%) resulted from high-energy trauma, including vehicular accidents or falls from heights. This finding differs significantly from the study by Kenmegne et al. (28), which reported that among 114 young patients with FNF, 99 cases (86.8%) were caused by high-energy trauma. However, Huang et al. (29) found that among 69 patients with FNF, 22 cases (31.9%) were attributed to high-energy trauma. This discrepancy may be related to differences in regional development levels. Nevertheless, it is undeniable that the incidence of FNF caused by high-energy trauma is on the rise.

During surgery, all cases met the reduction criteria of the Garden alignment index (17), achieving either Garden grade I or II. This study examined surgical time and incision length across both groups, which was consistent with previous studies, revealing no meaningful difference (*P* > 0.05). This result indicated that FNS and CCS exhibited similar outcomes regarding these complications, including minimally invasive characteristics and procedural simplicity. He et al. (30) reported an average surgical time of 49.9 min for FNS and 56.1 min for CCS; although FNS procedures required less time than CCS, the variation was not statistically meaningful. Conversely, Hu et al. (31) uncovered that the average surgical time for FNS was significantly greater than that for CCS, possibly attributed to differences in device design and surgeons' proficiency. Although there is currently no definitive evidence indicating that surgical time affects the prognosis of FNF, a shorter surgical duration is generally beneficial. Therefore, proficiency in using FNS is crucial for reducing surgery duration. In this study, the incision length for both surgical methods was approximately 4 cm, indicating minimal soft tissue disruption and adherence to the objectives of limited-access surgical strategies. Additionally, this finding indirectly demonstrated that comparable VAS outcomes were observed between both cohorts at 1 and 3 months after surgery.

The evaluation of the safety and efficacy of internal fixation primarily depends on three aspects: (1) the incidence of postoperative complications, (2) functional recovery of the hip (assessed by the HHS), and (3) fracture healing time. While CCS leads to minimal disruption to femoral neck vascularity and provides site-specific compression to promote fracture consolidation, its vertical shear resistance is relatively weak. Therefore, CCS can lead to screw loosening and femoral neck shortening postoperatively (31). Furthermore, once femoral neck shortening occurs, the screw loosening is likely to develop, which, if severe, can lead to hip pain or soft tissue irritation. FNS consists of four components: a dynamic rod, a steel plate, an anti-rotational element, along with a locking component. The anti-rotational and dynamic components, in combination with the locking screw, provide angular stability. Additionally, the anti-rotation screw interlocks with the dynamic rod, and its bifurcation enhances rotational stability. The most significant feature is that there is a 20 mm sliding compression space in the plate steel sleeve. Such a feature allows the dynamic rod to slide within the sleeve to achieve dynamic compression, thereby promoting fracture healing and preventing femoral neck shortening and screw loosening (13, 14). Earlier investigations have indicated that a reduction in femoral neck length exceeding 5 mm significantly decreases the quality of life of patients and affects hip joint function (32, 33). Therefore, in this study, femoral neck shortening exceeding 5 mm was classified as a postoperative complication. Notably, although FNS demonstrated clear advantages in fixation-related complications, no significant differences were observed between the FNS and CCS groups with respect to femoral head necrosis or nonunion. This finding may be attributed to the relatively small sample size and the limited follow-up duration, which may not fully capture late-onset complications. Longer follow-up with larger cohorts is needed to determine whether FNS confers any long-term advantage in these outcomes. Nonetheless, in the FNS group, both femoral shortening and screw breakage occurred in one case (4.2%). In contrast, the CCS group recorded six patients (27.3%) with femoral neck shortening and seven (31.8%) with screw loosening. The FNS group exhibited a significantly lower incidence of fixation-related issues like loosening, neck shortening, and screw breakage, relative to the CCS group, reaching statistical significance. Furthermore, the superiority of FNS over CCS in terms of angular and rotational stability can be further supported by previous biomechanical experimental results (13, 14) and the findings of this clinical study. Zielinski et al. demonstrated a relationship between femoral neck shortening and fracture classification (5). However, in this study, the two groups showed comparable fracture type distributions without statistically meaningful variation. Despite this, FNS-treated individuals exhibited fewer cases of femoral neck shortening. These findings mirror those reported by Weil et al. (34), suggesting that fracture type is not a major determinant of neck shortening.

In this study, HHS was used to assess the postoperative hip function recovery. At 1 month postoperatively, no notable intergroup difference was detected, whereas at 3 and 6 months, the FNS group exhibited significantly superior HHS scores compared to CCS. During postoperative rehabilitation, all patients were instructed to avoid load-bearing ambulation until fracture healing was achieved. Hence, hip joint function exhibited no marked difference between the two cohorts at 1 month, while partial loading was permitted starting at 3 months postoperatively. Thus, hip joint function was remarkably enhanced in the FNS group relative to the CCS at both 3 and 6 months postoperatively. Zlowodzki et al. (32, 33) discovered that femoral neck shortening could impair hip performance. Slobogean (35) reported that in patients under 55, marked femoral neck shortening post-fixation was associated with worse hip function at 12 months than in cases with minimal shortening. In this study, FNS-treated patients demonstrated superior hip function relative to the CCS-treated patients at both 3 and 6 months postoperatively. This may be attributed to the less frequent occurrence of femoral shortening in the FNS population compared to those receiving CCS, or the faster fracture consolidation observed in the FNS group. Additionally, FNS could more effectively promote fracture healing. In this trial, the mean fracture healing time was 4 months for FNS and 4.79 months for CCS, showing a statistically meaningful difference. This might be attributed to FNS facilitating dynamic compression at the fracture interface via its dynamic rod while effectively mitigating femoral neck shortening. In contrast, CCS failed to effectively prevent femoral neck shortening due to its weaker shear force resistance. This limitation is particularly evident in FNF with larger Pauwels angles, where screw loosening and micro-movements at the fracture site are more likely to occur. Consequently, the FNS group exhibited a markedly shorter fracture healing time. Although FNS offers greater mechanical stability, both groups followed the same weight-bearing protocol; thus, the shorter healing time in the FNS group is unlikely to result from earlier weight-bearing. This study demonstrates the advantages of FNS over CCS in the internal fixation of FNF. However, several limitations must be acknowledged. First, the retrospective and single-center design may have introduced selection bias, and the non-random group allocation inherent to retrospective studies may have further contributed to this issue. Second, the relatively small sample size and strict inclusion criteria may limit the generalizability of the findings. In addition, no formal sample size calculation was performed due to the retrospective design, which may reduce the statistical power to detect infrequent outcomes such as femoral head necrosis. Third, despite a follow-up duration of 9–24 months, the relatively short-term functional assessment may limit the detection of late-onset complications, as trauma research often recommends a minimum follow-up of at least 12 months. Variations in patients' pain sensitivity and adherence to rehabilitation could also have influenced VAS and HHS outcomes. Moreover, postoperative rehabilitation criteria were based on routine radiographic and clinical assessments without quantitative definitions, and outcome evaluation relied mainly on HHS and VAS rather than broader quality-of-life measures. Furthermore, as with all retrospective studies, residual confounding cannot be entirely excluded; unmeasured factors such as diabetes, osteoporosis, or other chronic conditions may still have affected outcomes despite comparable baseline characteristics. Smoking status was also not consistently available in the medical records, and its potential influence on fracture healing could not be fully assessed, representing an additional source of residual confounding. Finally, given the relatively short clinical history of FNS, longer follow-up is required to assess its long-term efficacy and durability. Therefore, future multicenter prospective studies with larger cohorts and extended follow-up are warranted to more comprehensively evaluate the long-term outcomes of FNS.

## 5 Conclusion

FNS facilitates bone healing, shortens healing time, and significantly reduces fixation-related postoperative complications, including femoral neck shortening, screw instability, and hardware failure, thereby improving early postoperative hip function and mobility. These findings indicate that FNS is a reliable and safe internal fixation option for the treatment of femoral neck fractures. Although this study included both Pauwels type II and III fractures, subgroup-specific differences could not be determined due to the limited sample size. Therefore, whether FNS confers differential benefits across Pauwels classifications warrants further investigation. As this study represents an initial, single-center experience, future multicenter studies with larger cohorts and longer follow-up periods are required to validate the long-term efficacy of FNS.

## Data Availability

The original contributions presented in the study are included in the article/Supplementary Material, further inquiries can be directed to the corresponding author.
