# Correction: Clinical application of the femoral neck system in the treatment of femoral neck fractures

**DOI:** 10.3389/fsurg.2026.1808979

**Published:** 2026-03-23

**Authors:** Guangyao Li, Xiaodan Xu, Junlong Song, Jinbo Liu, Qingsong Li, Zhenhai Pan, Weize Sun, Jingri Jin

**Affiliations:** 1 Medical College of Yanbian University, Yanbian, Jilin, China; 2 Department of Anesthesiology, Tai'an Eightty-Eight Hospital, Tai'an, Shandong, China; 3 Orthopedic Diagnosis and Treatment Center, Yanbian University Hospital, Yanbian, Jilin, China; 4 Department of Orthopedics, Traditional Chinese Medicine Hospital of Antu County, Yanbian, Jilin, China; 5 Joint Surgery Department, Tai'an Eightty-Eight Hospital, Tai'an, Shandong, China

**Keywords:** cannulated compression screw, femoral neck fracture, femoral neck system, internal fixation, young and middle-aged patients

Affiliation 2 “Department of Anesthesiology, Tai'an Eightty-Eight Hospital, Tai'an, Shandong, China” was erroneously given as “Operating Room of the Affiliated Hospital of Yanbian University, Yanbian, Jilin, China”.

Affiliation 5 “Joint Surgery Department of Tai'an Eightty-Eight Hospital, Tai'an, Shandong, China” was omitted from the paper. This affiliation has now been added for author Jingri Jin.

Author Jingri Jin was erroneously assigned to affiliation 3 “Orthopedic Diagnosis and Treatment Center, Yanbian University Hospital, Yanbian, Jilin, China”. This affiliation has now been removed for author Jingri Jin.

The original version of this article has been updated.

